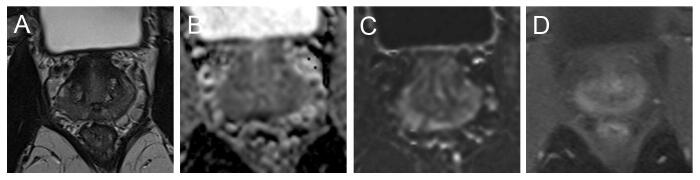# Characterisation of Multiparametric Magnetic Resonance Imaging of the Prostate in Younger Men with Normal Prostate-specific Antigen Within the PROBASE Study

**DOI:** 10.1016/j.euros.2025.03.014

**Published:** 2025-04-10

**Authors:** Rouvier Al-Monajjed, Lars Schimmöller, Jan Philipp Radtke, Jale Lakes, Agne Krilaviciute, Heinz-Peter Schlemmer, Kathleen Herkommer, Petra Seibold, Nikolaus Becker, Rudolf Kaaks, Boris Hadaschik, Gerald Antoch, Peter Albers, Matthias Boschheidgen

**Affiliations:** a University Dusseldorf, Medical Faculty, Department of Urology, D-40225 Dusseldorf, Germany; b University Dusseldorf, Medical Faculty, Department of Diagnostic and Interventional Radiology, Dusseldorf, D-40225 Germany; c Department of Diagnostic, Interventional Radiology and Nuclear Medicine, Marien Hospital Herne, University Hospital of the Ruhr-University Bochum, Herne, Germany; d Division of Personalized Early Detection of Prostate Cancer, German Cancer Research Center (DKFZ), Heidelberg; e Department of Radiology, German Cancer Research Center (DKFZ), Heidelberg; f Department of Urology, School of Medicine and Health, Technical University of Munich, Munich, Germany; g Division of Cancer Epidemiology, German Cancer Research Center (DKFZ), Heidelberg; h Department of Urology, University of Duisburg-Essen and German Cancer Consortium (DKTK), University Hospital Essen, Essen; i Center for Integrated Oncology Aachen Bonn Cologne Düsseldorf (CIO ABCD)

**Keywords:** Prostatic neoplasms, Multiparametric magnetic resonance imaging, Screening, Prostate-specific antigen

## Abstract

**Background and objective:**

Multiparametric magnetic resonance imaging (mpMRI) has emerged as an essential tool for the diagnosis of prostate cancer (PC). However, the right time to start screening for PC is not defined. This study aims to analyse mpMRI in young men at the age of 47–52 yr with normal prostate-specific antigen (PSA) values of ≤3 ng/ml.

**Methods:**

In this prospective analysis, consecutive men undergoing PSA screening with PSA levels below 3 ng/ml were offered mpMRI as part of the PROBASE study. Magnetic resonance imaging (MRI) parameters were assessed, and the findings included changes in T2-weighted (T2w) images, apparent diffusion coefficient (ADC), and dynamic contrast enhancement (DCE). Prostate Imaging Reporting and Data System (PI-RADS) category 4 or 5 would indicate a biopsy. The Kruskal-Wallis test was used to compare the ADC and PSA values across different PI-RADS categories, and the Spearman rho test was used to examine the relationship between T2w changes and PSA values for PI-RADS categories.

**Key findings and limitations:**

Forty-seven men were included (median PSA 1.22 ng/ml; interquartile range 0.47–1.79 ng/ml) between September 2021 and March 2022. High-quality MRI (median Prostate Imaging Quality [PI-QUAL] score 5) resulted in a median PI-RADS classification of 2 with low prostate volumes (median 27 ml). PI-RADS 3 classification occurred in 45% (median PSA 1.51 ng/ml). No score higher than PI-RADS 3 was observed. After 2-yr follow-up, no PC was reported in these men. For the peripheral zone (PZ), diffuse T2w changes were present in 81%. Focal and accentuated T2w changes were detected in 11% and 40%, respectively. DCE of the PZ was observed partially and severely in 53% and 17%, respectively. Limitations of this study are its small sample size, which provides more uncertainty regarding whether we were able to find reliable estimates for the outcomes explored in this study, the single-centre design, and the limited histopathological proof of nonmalignant cases.

**Conclusions and clinical implications:**

In younger men with normal PSA levels, clinically significant PC was not detected on MRI. When using the PI-RADS classification, extensive T2w changes and DCE led to a classification in category 3, an observation that we made frequently in this cohort. The MRI appearance in these cases seem more likely to be age specific or inflammatory due to not PC-specific ADC values, but nonetheless PC cannot be excluded safely.

**Patient summary:**

Magnetic resonance imaging characteristics in young healthy men with normal prostate-specific antigen levels are, in particular, diffuse T2-weighted hypointensity and dynamic contrast enhancement of the peripheral zone. These seem to be more likely age specific or inflammatory, but resulted in a higher proportion of patients with Prostate Imaging Reporting and Data System 3 classification.

## Take Home Message

The magnetic resonance imaging characteristics in young healthy men with normal prostate-specific antigen levels are, in particular, diffuse T2-weighted hypointensity and dynamic contrast enhancement of the peripheral zone. This seems more likely to be age specific or inflammatory, but resulted in a higher proportion of patients with Prostate Imaging Reporting and Data System 3 classification.

## 1 Introduction

Accurate diagnosis is crucial in men suspected of having prostate cancer (PC). A reliable diagnostic tool should effectively distinguish between benign and malignant conditions. Multiparametric magnetic resonance imaging (mpMRI) has emerged as an essential tool in the diagnostic pathway of PC and has become an integral part of international PC guidelines, enabling a magnetic resonance imaging (MRI)-guided biopsy to address suspicious targets detected on mpMRI [1], [2], [3], [4].

Recently, MRI has been incorporated into various screening strategies [5], [6], [7], [8], [9]. However, population-based screening remains unestablished, with many options available only through patient self-referral. In many screening strategies, MRI is used as a follow-up test when the initial screening, such as prostate-specific antigen (PSA) levels or other PC biomarkers, shows abnormal results. Owing to the modest sensitivity of PSA, international guidelines have been unable to provide a threshold recommendation. Some studies utilise MRI as the primary screening tool, potentially replacing PSA testing entirely (eg, IP1-PROSTAGRAM and MVP) [7], [8]. In this context, the precision of MRI is crucial. Another challenge is the correct image interpretation in the context of different age groups. Experience with imaging for screening in elderly men has already been reported (eg, Gothenburg and Stockholm studies) [5], [6]. Prostate Imaging Reporting and Data System (PI-RADS) version 2.1 was also originally designed and assessed for an older male population [10], [11], [12]. However, the extent to which this can also be transferred to younger collectives remains unclear. Data from the on-going “BLINDED” screening study demonstrated that interpretation of MRI results in young men (first screening round at 45 yr) is challenging and benefits significantly from expert reading [13].

There is a lack of information about the MRI appearance of ostensibly healthy younger men, with normal PSA levels and without any prostate clinic manifestations (such as florid inflammation, benign prostatic hyperplasia, and lower urinary tract symptoms). However, this knowledge is essential for the interpretation of changes in screening and for correct categorisation. Only a few studies have addressed the appearance of MRI in young men without PC in histopathology [14], [15], [16].

Considering the fact that the right time to start screening for PC is not defined, it is essential to investigate and also analyse MRI examinations in healthy young men. Therefore, with this study, we aimed to characterise typical MRI appearance in men at the age of 50 yr, since this is important for the understanding and interpretation of MRI examinations in this age group and provides further information, especially when considering MRI as part of future screening strategies at a population level.

## 2 Patients and methods

### 2.1 Study population and design

This study (MRInPSA) was designed for men within the PROBASE trial [17]. The study was approved by the local ethics committee. Written informed consent was obtained from every participant. Between September 2021 and March 2022, men invited to PROBASE underwent PSA screening. PROBASE analyses the benefits of PSA screening starting from the age of 45 or 50 yr and offers mpMRI of the prostate to men with elevated PSA values above 3.0 ng/ml. However, as part of this study, men at the age of 47–52 yr without an elevated PSA level were also invited for an MRI examination. The first screening visit with the determination of PSA blood levels was about 8 wk before the MRI examination. Confirmatory PSA values were determined after an interval of 4 wk from the first screening visit. All the included men were therefore categorised as having a low risk. We systematically evaluated MRI data (prostate volume, PSA density [PSAD], image quality, PI-RADS category, diffuse signal changes in the peripheral zone [PZ], presence of focal changes in the PZ, representative highest and lowest apparent diffusion coefficient [ADC] values, enhanced signal at high b value images, changes in dynamic contrast enhancement [DCE], and presence of hyperplasia). We focused, in particular, on the characteristics of the PZ, as this is where the majority of clinically significant tumours arise, and this requires special attention in young men, whereas in older men, the transition zone also comes more into focus due to the presence of benign prostate hyperplasia. For a further analysis for distinguishing between different PI-RADS categories, ADC and PSA were the only metrics tested because these were considered the most biologically and clinically relevant markers. This decision was made a priori and based on their established role in PC detection, PSA as a widely used serum biomarker and ADC as a key imaging parameter reflecting PC cellularity. MR image quality was assessed with the Prostate Imaging Quality (PI-QUAL) score [18]. All patients were followed up over a period of at least 2 yr by means of PSA monitoring and repeat MRI in the event of PI-RADS 3 findings or an increase in PSA. The study objective was to quantify parameters, which characterise MRI in this specific age group to better understand the MRI appearance in younger men and to improve the reading of prostate MRI in an early screening setting.

### 2.2 Imaging

All mpMRI scans were conducted u 3 T MRI scanners. MRI parameters contained T2-weighted (T2w) turbo spin echo sequences in three planes (axial: voxel size 0.5 × 0.5 × 3.0 mm^3^; field of view 130 mm), diffusion-weighted imaging (DWI; z-EPI [ZOOMit; Siemens Healthineers] and rs-EPI [RESOLVE; Siemens Healthineers]; voxel size 1.4 × 1.4 × 3.0 mm^3^; b values 0, 500, and 1000 s/mm^2^ plus calculated 1800 s/mm^2^), and DCE (T1 vibe; voxel size 0.8–1.5 × 0.8–1.5 × 3.0 mm^3^, scan time 3 min, temporal resolution 7 s) in line with the recommendations of PI-RADS version 2.1 [19], [20]. ADC parameter maps were calculated by the scanner using the standard monoexponential model.

### 2.3 Data and image analysis

**Fig. 1:**
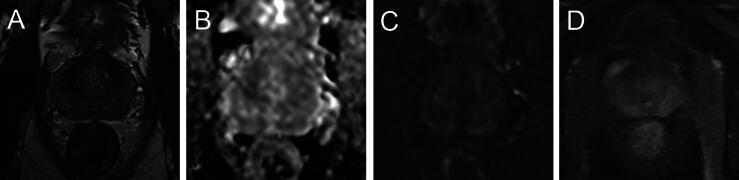
For 50-yr-old men, PSA 0.39 ng/ml (PSAD 0.01 ng/ml/cm^3^). Multiparametric MRI with (A) severe diffuse but no focal T2w changes in the peripheral zone (PZ), (B) no focal changes on ADC map or (C) high b-value images, and (D) slight symmetric enhancement on DCE in PZ, PI-RADS 3, and PI-QUAL 4. ADC = apparent diffusion coefficient; DCE = dynamic contrast enhanced imaging; MRI = magnetic resonance imaging; PI-QUAL = Prostate Imaging Quality; PI-RADS = Prostate Imaging Reporting and Data System; PSA = prostate-specific antigen; PSAD = PSA density; PZ = peripheral zone; T2w = T2 weighted.

**Fig. 2:**
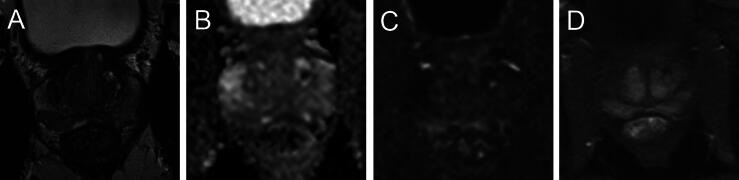
For 51-yr-old men, PSA 2.36 ng/ml (PSAD 0.04 ng/ml/cm^3^). (A) Multiparametric MRI with partial diffuse and focal T2w changes in the peripheral zone (PZ), (B) diffuse changes on ADC map and (C) high b-value images, and (D) symmetric enhancement on DCE in PZ, PI-RADS 3, and PI-QUAL 5. ADC = apparent diffusion coefficient; DCE = dynamic contrast enhanced imaging; MRI = magnetic resonance imaging; PI-QUAL = Prostate Imaging Quality; PI-RADS = Prostate Imaging Reporting and Data System; PSA = prostate-specific antigen; PSAD = PSA density; PZ = peripheral zone; T2w = T2 weighted.

**Supplementary Fig. 2:**
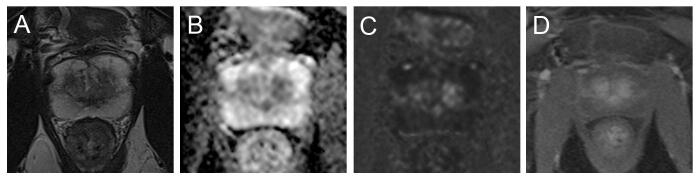


The mpMRI data were initially read and supervised by an uroradiologist (L.S.) with experience in reading prostate MRI of >10 000 cases as part of the clinical routine. Prostate volume was measured by dedicated software volumetry (DynaCAD; Philips Healthcare), and PSAD was calculated by dividing PSA blood levels by prostate volume. All examinations were evaluated following PI-RADS version 2.1, and the PI-RADS category was assessed for every study participant. The images were analysed again as part of the dedicated study evaluation by two board-certified radiologists (M.B. and L.S.) in consensus regarding MR characteristics on T2w imaging, DWI, and DCE. The decision regarding the rating was made by consensus. All images were evaluated regarding the presence of diffuse PZ hypointensity at T2w imaging, T2w focal changes, and enhancement pattern on DCE. DCE in the PZ was defined as positive if there was diffuse and early contrast enhancement (partial with 30–70% and severe with >70% involvement of the PZ). For DWI, regions of interest (ROIs) were defined on ADC maps. A circular ROI was placed in a representative PZ area with the highest ADC value and an ROI in an area with the lowest ADC value for each prostate individually. Representative ROIs did not include focal changes, even if any of these were detected on T2w images. Signal intensities were measured based on ROIs using the b1800 images. High b-value images were checked for high signal intensity areas and considered as positive if focal enhanced areas were present in the PZ. Examples for different MRI characteristics are given in Fig. 1, Fig. 2, and Supplementary Figs. 1 and 2.

### 2.4 Statistical analysis

Data are presented as median plus interquartile range (IQR) for both normal distributed variables (T2w hypointensity, DCE, and ADC) and non-normal distributed variables (age, PSA, PSAD, PI-RADS, and prostate volume). Statistical analyses were performed using IBM SPSS Statistics (version 27; IBM Corp, Armonk, NY, USA). We used the Kruskal-Wallis test to compare the ADC and PSA values across different PI-RADS categories because the data did not follow a normal distribution, making this nonparametric test appropriate for detecting differences between multiple independent groups. We applied the Spearman rho test to examine the relationship between the extent of diffuse or focal T2w changes and PSA values across PI-RADS categories as it is a nonparametric test suitable for assessing the correlations between ordinal and continuous variables without assuming a normal distribution.

## 3 Results

### 3.1 Participant characteristics

Fifty consecutive men (in the second screening round of the PROBASE study at 47–52 yr of age) were screened, all exhibiting PSA levels <3 ng/ml. Three men were excluded (one due to claustrophobia and two due to refusal after inclusion) so that our final collective consisted of 47 participants. Clinical characteristics are described in Table 1. None of the patients received a subsequent biopsy. After a minimum follow-up of 2 yr (median 38 mo, IQR 32–43 mo), no PC was detected in this cohort.

**Table 1:** Baseline parameters

Participants (*n*)	47
Age (yr), median (IQR)	50 (50–51)
PSA (ng/ml), median (IQR)	1.22 (0.47–1.69)
Prostate volume (ml), median (IQR)	27 (23–32)
PSAD, median (IQR)	0.04 (0.02–0.06)

IQR = interquartile range; PSA = prostate-specific antigen; PSAD = prostate-specific antigen density.

### 3.2 MRI characteristics

MRI characteristics are illustrated in Table 2. The median PI-RADS classification score was 2 (2–3) and the median PI-QUAL score was 5 (4–5). We detected diffuse PZ T2w hypointensity in over 80% of all men. Regional T2w changes were present in 40% (accentuated) and 11% (focal) of men. For DCE images, we observed diffuse and early enhancement patterns in 53% (partial) and 17% (severe), respectively. Regarding ADC values in DWI, we observed a broad distribution for the representative ROIs, with four men showing areas with low ADC values below 1000 (×10^−3^ mm^2^/s). Of the men, 30% had enlarged prostate volume of ≥30 ml; three of them exhibited a volume of ≥50 ml. We found a significant difference for PI-RADS categories in PSA levels (PSA cut-off 1.5 ng/ml; Table 3). Taking a closer look at PSA and ADC values, there was one patient with PI-RADS 1 category who showed a PSA value of 0.27, a low ADC value of 1762 × 10^−3^ mm^2^/s, and a high ADC value of 2017 × 10^−3^ mm^2^/s. PSA values and representative low ADC values differed significantly between the different PI-RADS categories of 2 and 3 (*p* = 0.029 and *p* = 0.021, respectively). The results are presented in Table 4.

**Table 2:** MRI characteristics

PI-RADS v2.1, *n* (%)		PI-RADS 1	1 (2.1)
		PI-RADS 2	25 (53)
		PI-RADS 3	21 (45)
PI-QUAL v1, *n* (%)		PI-QUAL 3	6 (13)
		PI-QUAL 4	8 (17)
		PI-QUAL 5	33 (70)
PZ	Diffuse T2w changes, *n* (%)	No	9 (19)
		Partial	29 (62)
		Severe	9 (19)
	Focal T2w changes, *n* (%)	No	23 (49)
		Accentuated	19 (40)
		Focal	5 (11)
	DCE, *n* (%)	No	14 (30)
		Partial	25 (53)
		Severe	8 (17)
	ADC values (×10^−3^ mm^2^/s), median (IQR)	High	1827 (1692–1977)
		Low	1185 (1096–1316)
	DWI positive, *n* (%)		8 (17)

ADC = apparent diffusion coefficient; DCE = dynamic contrast enhancement DWI = diffusion weighted imaging; IQR = interquartile range; PI-RADS = Prostate Imaging Reporting and Data System; PI-QUAL = Prostate Imaging Quality; PZ = peripheral zone; T2w = T2-weighted imaging.

**Table 3:** Comparison of PI-RADS, PSA, and T2w changes

		Diffuse T2w changes in PZ			*p* value
		No	Partial	Severe	
PI-RADS	1	1	0	0	0.25
	2	4	18	3	
	3	4	11	6	
		Focal T2w changes in PZ			
		No	Accentuated	Focal	

PI-RADS	1	1	0	0	0.067
	2	16	8	1	
	3	6	11	4	
		PSA			
		<1.5 ng/ml		≥1.5 ng/ml	

PI-RADS	1	1		0	**0.021**
	2	20		5	
	3	10		11	

PI-RADS = Prostate Imaging Reporting and Data System; PSA = prostate-specific antigen; PZ = peripheral zone; T2w = T2-weighted imaging.

**Table 4:** Comparison of PI-RADS category with PSA and ADC values

	PI-RADS 2 (*n* = 25)	PI-RADS 3 (*n* = 21)	*p* value
PSA (ng/ml), median (IQR)	0.84 (0.45–1.42)	1.51 (1.05–2.17)	**0.029**
ADC low (×10^−3^ mm^2^/s), median (IQR)	1242 (1150–1429)	1136 (1065–1209)	**0.021**
ADC high (×10^−3^ mm^2^/s), median (IQR)	1779 (1730–1951)	1817 (1622–1882)	0.3

ADC = apparent diffusion coefficient; IQR = interquartile range; PI-RADS = Prostate Imaging Reporting and Data System; PSA = prostate-specific antigen.

## 4 Discussion

Diffuse changes on MRI present a diagnostic challenge, particularly in the context of young men undergoing MRI in a screening setting. Understanding the influence of MRI on sensitivity and specificity in this specific male population is essential. This study of healthy men with PSA values below 3 ng/ml and a median age of 50 yr showed overall a not PC-specific MRI appearance but still resulted in a high proportion of patients with PI-RADS 3 classification. The higher representative ADC values of our cohort than those of the PC cases in the first screening cohort of the PROBASE trial of 50-yr-old men with PSA ≥3 ng/ml and that no case was classified to have PI-RADS 4 or 5 indicate a collective most likely without clinically significant PC (csPC), although the present study lacks a histopathological correlation [13].

A study by Nam et al [8] comparing MRI and PSA screening included approximately 18% of men with csPC and normal PSA values. As there are rare subtypes of PC without PSA expression (eg, neuroendocrine PC), csPC cannot be ruled out by low PSA alone [21]. However, these csPC cases will be discovered with high certainty by MRI (PI-RADS 4 or 5). However, the high incidence of PI-RADS 3 findings is still striking. Similarly, over 40% of the first PROBASE cohort screened with mpMRI was reported to have PI-RADS 3 at reference reading. In this group with PSA values ≥3 ng/ml, in 11% csPC was identified on a subsequent MRI/transrectal ultrasound–fusion biopsy [13]. In the Goteborg study, targeted biopsies for PI-RADS 3 yielded 23% International Society of Urological Pathology (ISUP) 1 cancers and only 2% more aggressive tumours (2% ISUP3 and none ISUP 4/5) [5]. In our study, men exhibited an even higher prevalence of PI-RADS 3 findings (45%). In our cohort, especially those with PSA levels below 1.5 ng/ml showed inconspicuous MRI findings, indicating a highly unlikely presence of clinically relevant PC in these cases. However, it is worth noting that the cases with PI-RADS 3 had a significantly higher PSA value. It is known that the prevalence of PC rises with increasing PSA values, starting below 3 ng/ml [22]. Therefore, for this group, it is difficult to rule out PC with certainty, particularly because these PI-RADS 3 cases showed more extensive T2w changes with a corresponding subtle ADC reduction. However, evaluation of participants’ medical history in the PROBASE database with now more than 2 yr of follow-up has shown no PC cases yet. A possible approach to handle PI-RADS 3 cases in screening scenarios could primarily be a follow-up with MRI and PSA testing without a biopsy to avoid overdiagnosis. A biopsy might be offered only in cases with elevated PSAD (≥0.15 ng/ml/cm^3^). The PROBASE study will give more clear answers on that.

Analogous to previous studies, we also observed diffuse T2w signal changes in the PZ [14], [15], [16]. These areas of hypointensity align with the findings of De Visschere et al [23], where areas of hyperintensity were considered “normal” in older men, indicative of glandular atrophy. The ADC values in atrophic areas were higher, which was attributed to the higher water or mucinous content between the atrophic tissues. Since diffuse T2w changes in the PZ seem more often in younger men, atrophic appearance at MRI would be considered more often in older men [17]. This is consistent with the observations made by Bura et al [15], who illustrated that younger males without PC displayed diminished T2w signal intensities in the PZ, diffuse enhancement on DCE imaging and reduced ADC values. A hypothesised correlation between age and the background heterogeneity of the PZ was proposed.

A diverse spectrum is observed in young men who exhibit a range of conditions, including inflammation and, in some cases, early atrophy within the PZ. Still, T2w hypointensity cannot causally be linked to inflammation or tumour. Instead, it should be regarded as age-specifically normal, rendering tumour differentiation more complex. Other sequence measurements must be employed to assist in this regard, with the ADC appearing particularly helpful. The potential implications of our data on the characterisation of MRI alterations in individuals with benign histopathological reports need further evaluation in subsequent clinical trials. Benign disease, such as prostatitis and atrophy, can mimic PC on MRI [19], [24], [25], [26], [27], [28]. DWI, and especially ADC, is one marker for differentiating between PC and inflammatory disease. Uysal et al [25] stated ADC values higher than 955 (×10^−3^ mm^2^/s) to predict noncancerous histopathology. In our collective, we found rather high ADC values overall, also in comparison with the negatively biopsied patients in the first round of screening as part of the BLINDED study, with PSA values above 3 ng/ml. Here, the median ADC value was 1000 (×10^−3^ mm^2^/s) [28]. In fact, the ADC values observed here are more in the range of the values of a previously published collective, in which atrophy and prostatitis could be detected histologically; tumours were not detected here [14]. Nevertheless, PI-RADS 3 cases had significantly lower ADC values, which again emphasises the difficulty of differentiating carcinomas.

Discussion of the results in the context of screening for PC raises the question whether MRI is of value under these circumstances. MRI screening requires significant expertise, especially when interpreting scans without contrast agents. Widespread uses in screening programmes are currently limited by infrastructure and economic constraints, particularly in Europe and other regions. Furthermore, the high rates of PI-RADS 3 cases would result in high recall rates with even more MRI examinations. A potential solution lies in the widespread implementation of strictly certified high-volume diagnostic centres. In addition, it must also be discussed whether the PI-RADS classification is adequate for MRI reading in a screening setting. PI-RADS classification is primarily based on experiences in significantly older collectives, in which the image impressions differ from the screening MRI in young men. Adaptions of the MRI scoring, which, for example, set a higher threshold value for biopsy decision and/or report a positive finding only in the case of clear tumours, may therefore be useful [9], [29].

This study has limitations. First, the study is limited by its sample size and the single-centre evaluation. Second, we have no histopathology as proof for nonmalignant cases. However, there was neither PI-RADS 4 nor PI-RADS 5 classification in the cohort with a median PSAD of 0.04 ng/ml/cm^3^ and no reported PC case, within the PROBASE database, among men analysed in our subproject during the follow-up of 2 yr. Still, a 2-yr follow-up might be insufficient to securely rule out tumour, given the natural history of PC.

## 5 Conclusions

Our study characterises the imaging appearance of MRI in younger men with PSA values below 3 ng/ml. Characteristics are diffuse T2w hypointensity, DCE of the PZ, and mean ADC between 1200 and 1800 (×10^−3^ mm^2^/s). Nearly half of all participants were classified as having PI-RADS 3, emphasising the difficulties of applying the PI-RADS system to younger individuals in a screening context. High expertise in the interpretation of MRI results might be helpful in addressing this issue. Even if no tumour could be detected at follow-up after 2 yr, the detection rates of PI-RADS category 3 in the PROBASE study in young men with PSA ≥3 ng/ml raise the question whether there are also (undetected) PC in PI-RADS 3 cases with PSA below 3 ng/ml.

  ***Author contributions*:** Lars Schimmöller had full access to all the data in the study and takes responsibility for the integrity of the data and the accuracy of the data analysis.

  *Study concept and design*: Schimmöller, Boschheidgen, Radtke, Al-Monajjed, Albers.

*Acquisition of data*: Lakes, Boschheidgen, Krilaviciute, Schimmöller, Schlemmer.

*Analysis and interpretation of data*: Al-Monajjed, Boschheidgen, Radtke, Schimmöller.

*Drafting of the manuscript*: Al-Monajjed, Boschheidgen, Schimmöller.

*Critical revision of the manuscript for important intellectual content*: Schimmöller, Lakes, Krilaviciute, Schlemmer, Herkommer, Hadaschik, Seibold, Becker, Kaaks, Antoch, Albers.

*Statistical analysis*: Al-Monajjed, Boschheidgen, Radtke, Schimmöller.

*Obtaining funding*: Schimmöller, Herkommer, Hadaschik, Seibold, Becker, Kaaks, Antoch, Albers.

*Administrative, technical, or material support*: None.

*Supervision*: Schimmöller, Boschheidgen, Al-Monajjed, Albers.

*Other*: None.

  ***Financial disclosures:*** Lars Schimmöller certifies that all conflicts of interest, including specific financial interests and relationships and affiliations relevant to the subject matter or materials discussed in the manuscript (eg, employment/affiliation, grants or funding, consultancies, honoraria, stock ownership or options, expert testimony, royalties, or patents filed, received, or pending), are the following: None.

  ***Funding/Support and role of the sponsor*:** The PROBASE trial is funded by the Deutsche Krebshilfe (Stiftung Deutsche Krebshilfe, DKH).

## Figures and Tables

**Supplementary Fig. 3:**