# Supplementary material for: Characterisation of Multiparametric Magnetic Resonance Imaging of the Prostate in Younger Men with Normal Prostate-specific Antigen Within the PROBASE Study
Source: Eur Urol Open Sci. 2025 Apr 10;75:94–100. doi: 10.1016/j.euros.2025.03.014 (PMC12008618; doi:10.1016/j.euros.2025.03.014)
Supplement: Supplementary Data 1 [file mmc1.docx]

**Supplementary Figure 1:** 50 year old men, PSA 1.42 ng/ml (PSAD 0.03 ng/ml/cm^3^). Multiparametric MRI with no diffuse or focal T2w changes in the peripheral zone (PZ) (A), no focal changes on ADC map (B) or high b-value images (C), and enhancement on DCE in PZ (D), PI-RADS 2, PI-QUAL 5.

**Supplementary Figure 2:** 52 year old men, PSA 1.71 ng/ml (PSAD 0.08 ng/ml/cm^3^). Multiparametric MRI with severe diffuse and accentuated focal T2w changes in the peripheral zone (PZ) (A), diffuse changes on ADC map (B) and high b-value images (C), and accentuated focal diffuse enhancement on DCE in PZ (D), PI-RADS 3, PI-QUAL 4.
